# Abrasion-Induced Acceleration of Melt Crystallisation of Wet Comminuted Polybutylene Terephthalate (PBT)

**DOI:** 10.3390/polym14040810

**Published:** 2022-02-19

**Authors:** Florentin Tischer, Björn Düsenberg, Timo Gräser, Joachim Kaschta, Jochen Schmidt, Wolfgang Peukert

**Affiliations:** 1 Institute of Particle Technology, Friedrich-Alexander-Universität Erlangen-Nürnberg, Cauerstraße 4, D-91058 Erlangen, Germany; florentin.riedel@fau.de (F.T.); bjoern.duesenberg@fau.de (B.D.); timo.graeser@fau.de (T.G.); jochen.schmidt@fau.de (J.S.); 2 Institute of Polymer Materials, Friedrich-Alexander-Universität Erlangen-Nürnberg, Martensstraße 7, D-91058 Erlangen, Germany; joachim.kaschta@fau.de

**Keywords:** wet comminution, stirred media mills, abrasion, polymer, additive manufacturing, powder bed fusion, thermal properties, isothermal DSC

## Abstract

Within this contribution, the effect of grinding media wear on the melt crystallisation of polybutylene terephthalate (PBT) is addressed. PBT was wet ground in a stirred media mill in ethanol using different grinding media beads (silica, chrome steel, cerium-stabilised and yttrium-stabilised zirconia) at comparable stress energies with the intention to use the obtained particles as feed materials for the production of feedstocks for laser powder bed fusion additive manufacturing (PBF-AM). In PBF-AM, the feedstock’s optical, rheological and especially thermal properties—including melt crystallisation kinetics—strongly influence the processability and properties of the manufactured parts. The influence of process parameters and used grinding media during wet comminution on the optical properties, crystal structure, molar mass distribution, inorganic content (wear) and thermal properties of the obtained powders is discussed. A grinding media-dependent acceleration of the melt crystallisation could be attributed to wear particles serving as nuclei for heterogeneous crystallisation. Yttrium-stabilised zirconia grinding beads proved to be the most suitable for the production of polymer powders for the PBF process in terms of (fast) comminution kinetics, unchanged optical properties and the least accelerated crystallisation kinetics.

## 1. Introduction

Powder bed fusion of polymers with laser beam (PBF-LB/P) is a layer-by-layer additive manufacturing (AM) technique that allows for the production of complex 3D structured parts. Therefore, no tools or moulds are needed in comparison to conventional subtractive manufacturing processes [1,2,3,4].

A plastic powder suitable for the PBF process must meet specific intrinsic and extrinsic properties [2,5]. Intrinsic properties include optical and thermal properties such as sintering window, crystallisation kinetics or melt viscosity. Extrinsic properties include bulk powder properties such as flowability, bulk density, particle size distribution and particle shape. The dependencies of PBF processability and the resulting part properties on powder feedstock characteristics are the subjects of various papers and will not be addressed here in detail. A comprehensive discussion can be found, e.g., in [2,3,6,7,8,9]. A good PBF powder should be a semi-crystalline polymer with a wide sintering window, a low melt viscosity and slow crystallisation kinetics. An understanding of the isothermal crystallisation behaviour is especially important to prevent curling, i.e., the premature isothermal crystallisation of molten polymer during the build process [10]. For advantageous flowability and packing behaviour, the particles should be of rounded shape, have a narrow particle size distribution and a low fines content (<20 µm) [9,11].

Today, these powders are produced either by a liquid–liquid phase separation and precipitation process, as in the case of most PA12 powders [12,13], or by cryogenic comminution, as in the case of PA11 Rilsan Invent Natural (Arkema) [14]. However, the process of the cold-wet comminution of polymers in stirred media mills [15] shows much potential for the production of polymer powders for the PBF process. Compared to cryogenic (dry) comminution [16,17,18], no extreme temperatures are required, and it is possible to produce a wide range of different polymers in suitable sizes for the PBF process [15,19,20,21,22,23,24]. In particular, wet comminution allows one to produce smaller particles than dry comminution, in several cases even below 5 µm [15], which opens new possibilities for the production of novel composite powders or fine-structured parts using smaller than ‘state-of-the art’ particles, if the powder flowability is maintained. With an additional thermal rounding step, the flowability and bulk density of powders obtained by comminution can be further improved [20,25]. This makes the cold wet comminution process a suitable candidate for extending the limited commercially available range of powder feedstocks for PBF-AM [1,2]. So far, there are hardly any studies on the influence of the grinding media material on the intrinsic properties of the obtained wet-ground polymer particles. As the powder properties are governed by the process, the influence on the intrinsic properties of the polymers, such as isothermal crystallisation behaviour, should not be neglected.

In this work, the effect of grinding bead material on the comminution kinetics, optical properties, crystal structure, molar mass distribution, inorganic content (wear) and thermal properties of wet comminuted PBT is investigated. PBT was chosen as the semi-crystalline feed polymer due to its better thermal stability, low moisture absorption and higher mechanical strength than polyamides [26]. This could expand the range of applications for PBF-LB/P manufactured components for the electronics and automotive sectors, where higher thermal resistance or electrically insulating properties are required. The main focus is on the evaluation of the crystallisation behaviour, as expressed by the isothermal crystallisation kinetics and the width of the so-called sintering window. By ruling out changes in crystal structure and molar mass, the observed altered crystallisation behaviour could be solely assigned to be attributed to grinding media abrasion. Finally, we evaluate which grinding beads are best suited to the production of polymer powders for the PBF-LB/P process.

## 2. Theory

### 2.1. Wet Comminution in Stirred Media Mills

In cold wet comminution of polymers in stirred media ball mills, the polymer is comminuted by grinding beads in a liquid solvent. Therefore, the energy input takes place via the stirrer. The influence of the solvent in wet comminution is the subject of current research [27], but has hardly been investigated for polymers. Kwade [28] describes the comminution in stirred media mills via the mass-specific energy input *E_M_*. The mass-specific energy input *E_M_* can be calculated from the product of the average number of stress events *SN* and the stress energy *SE* of these events (Equation (1)).

(1) $$E_{M}=SN\cdot SE$$

For batch comminution, *SN* (Equation (2)) can be described by the number of impacts between the grinding media *N_C_*. *P_S_* is the probability that a particle is significantly stressed during an impact and *N_P_* is the total number of particles in the medium.

(2) $$SN=\frac{N_{C}P_{S}}{N_{P}}$$

A detailed derivation of the Equations used for the average number of sufficient stress events *SN* (Equation (3)) can be found in the literature [28,29,30,31]. Since the parameter filling ratio φ, solids concentration of the suspension *c_V_* and bulk porosity ε were kept constant in this work, the reduced stress number *SN_Red_* (Equation (4)) was used. The reduced stress number is only a function of the changed variables’ rotational speed *n*, process time *t*, grinding media diameter *d_GM_* and a characteristic particle size of the feed *x_Feed_* [28,31]. In this work, the volume median particle size *x*_50.3_ of the feed particles was used as the characteristic particle.

(3) $$SN\propto \;\frac{\varphi_{GM}\;\cdot \;\left(1-\varepsilon \right)}{\left(1-\varphi_{GM}\;\cdot \;\left(1-\varepsilon \right)\right)\;\cdot \;c_{V}}\;\cdot \;\frac{n\;\cdot t}{d_{GM}}$$

(4) $$SN_{Red}\propto \;n\cdot t\cdot {\left(\frac{x_{Feed}}{d_{GM}}\right)}^{2}$$

The stress energy *SE* is proportional to the maximum energy *SE_max_* (Equation (5)), which can be transferred during an impact between two grinding beads. For stirred media mills, this is the kinetic energy of two grinding media colliding with the speed of agitation *v_tip_*. Becker et al. [32] extended this formula by the elastic moduli of the grinding media and the feed particles to take into account the deformation of the grinding media, which reduces the transferred energy. This plays a minor role in polymer wet comminution since polymers have a significantly lower Young’s modulus than ceramics or steel [32], making the factor $\left(\frac{E_{GM}}{E_{Feed}+E_{GM}}\right)$ become 1 [13]. By using *SN_Red_* and *SE_max_*, a reduced mass-specific energy input *E_M,Red_* can be calculated using Equation (6).

(5) $$SE\propto SE_{max}=d_{GM}^{3}\cdot \rho_{GM}\cdot v_{tip}^{2}\cdot \left(\frac{E_{GM}}{E_{Feed}+E_{GM}}\right)$$

(6) $$E_{M,Red}\propto \;SN_{Red}\cdot SE_{max}=\rho_{GM}\cdot d_{GM}\cdot \pi^{2}\cdot d_{tip}^{2}\cdot n^{3}\cdot x_{Feed}^{2}\cdot t\;$$

### 2.2. Polymer Melt Crystallisation

**Figure 1:**
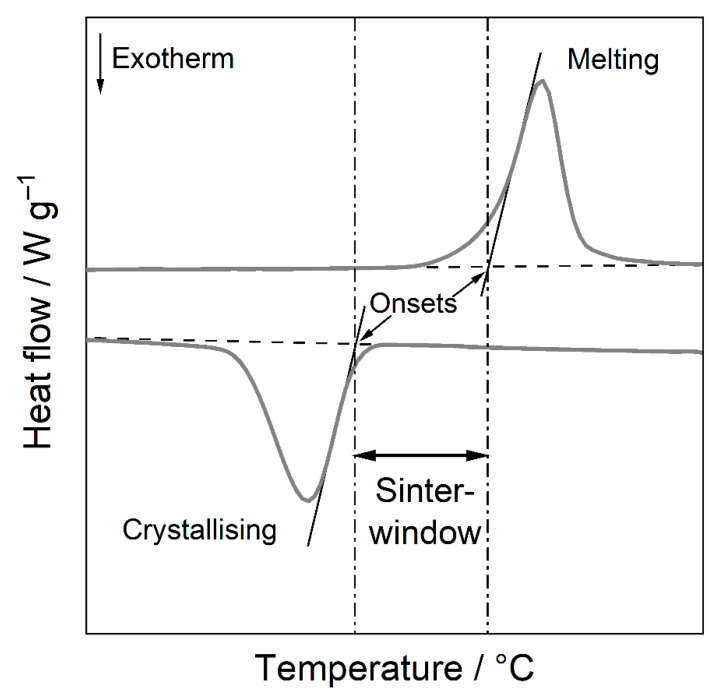
Schematic representation of the sintering window of thermoplastics defined as the area between the onset of crystallisation and the onset of melting.

The crystallisation of polymers plays a decisive role in PBF-LB/P. The onset of crystallisation defines the lower limit of the so-called sintering window (Figure 1), which is often used to determine the building chamber temperature.

From an economic point of view, in PBF/LB-P, the lowest possible process temperature is to be favoured in order to keep the production costs low (cf. lower energy consumption, less ageing of the overflow powder and part cake material, smaller build time). However, low building room temperatures result in a higher supercooling of the melt, which results in faster isothermal crystallisation of the polymer. Due to the volume shrinkage during crystallisation, warpage and curling can occur, if crystallisation is too fast. It is, therefore, of great importance to analyse and understand the crystallisation of the used polymer in order to be able to find optimal process parameters [33,34].

Crystallisation of polymers can be divided into two main steps: nucleation and crystal growth. Since nucleation plays an important role in this work to explain the changed thermal properties of the PBT powder, it will be briefly addressed. The solid and liquid phase are in equilibrium at the equilibrium melting temperature $T_{M}^{0}$. At this temperature, the change in free energy of melting Δ*G_M_* equals 0, see Equation (7) [35].

(7) $$\Delta G_{M}=\Delta H_{M}-T_{M}^{0}\cdot \Delta S_{M}=0$$

**Figure 2:**
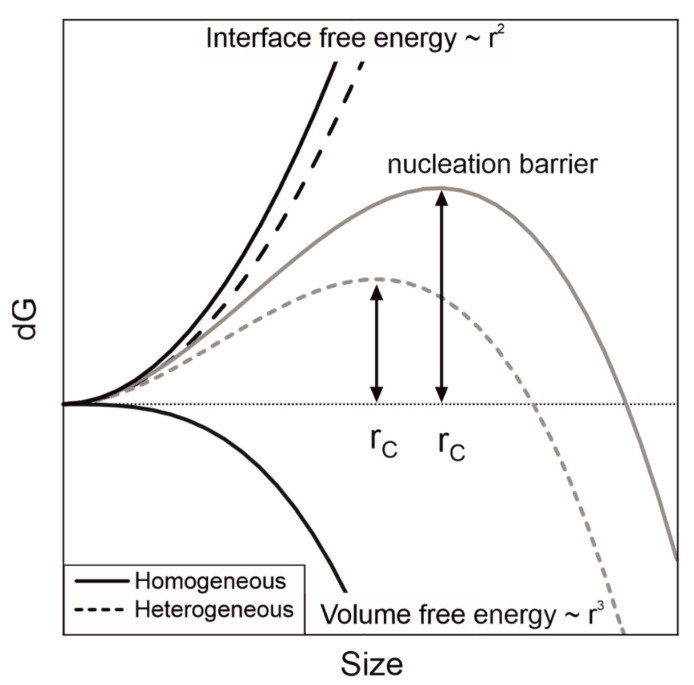
Schematic illustration of free energy as a function of nucleus size. For heterogeneous nucleation, less interface needs to be created, which lowers the nucleation barrier.

Below the equilibrium melting temperature, the solid phase is the thermodynamically more stable phase and the liquid phase, therefore, should undergo a phase transition. However, crystallisation is kinetically hindered and only starts, if the nucleation barrier can be overcome (Figure 2). Here, supercooling Δ*T* is the driving factor (Equations (9) and (10)). Nucleation is the first phase of crystallisation and consists of the three types, primary, secondary and tertiary nucleation. In primary nucleation, a new nucleus and, thus, a new surface are created. According to classical nucleation theory, this nucleus is only stable at a sufficient size, where the decrease in volume free energy (Δ*g∙V*) is greater than the increase in interface free energy (*σ∙A*), cf. Equation (8) [36].

(8) ΔG=σ·A−Δg·V

(9) $$\Delta g=\Delta h-T_{C}\cdot \Delta s\;\approx \;\Delta h-T_{C}\cdot \frac{\Delta h}{T_{M}^{\;}}=\Delta h\;\frac{T_{M}^{\;}-T_{C}}{T_{M}^{\;}}\;\propto \;\Delta T$$

(10) $$\Delta T=T_{M}^{0}-T_{C}$$

Primary nucleation can be distinguished between homogeneous and heterogeneous nucleation. Heterogeneous nucleation occurs at a foreign surface, such as seed particles, debris or adjacent walls. Compared to homogeneous nucleation, the energy barrier for heterogeneous nucleation is much lower and, therefore, nucleation sets in at lower supersaturation / lower supercooling compared to homogenous nucleation. Comparable to heterogeneous nucleation, secondary nucleation refers to the creation of a new nucleus but on one side of an existing nucleus and not on a foreign surface. Therefore, the resulting energy barrier is lower than primary homogeneous nucleation [35].

## 3. Materials and Methods

### 3.1. Materials

Polybutylene terephthalate (PBT RXP 7103 Natural, Resinex, Zwingenberg, Hesse, Germany) was sieved to remove the coarse material. The feed material used for the comminution experiments was characterized by *x*_10,3_ = 150 µm, *x*_50,3_ = 352 µm and *x*_90,3_ = 672 µm. Denatured ethanol (99%, VWR, PA, USA)) was used as solvent. Spherical grinding beads (2 mm) made from glass (Diamond Perls, Mühlmeier, Bärnau, Bavaria, Germany), yttrium-stabilised zirconia (ZY S, SiLi, Warmensteinach, Bavaria, Germany), cerium-stabilised zirconia (ZC-L, Mühlmeier) and chrome steel (W 1.3505, Mühlmeier), respectively, were used as grinding media (see Table 1).

### 3.2. Comminution Experiments

All comminution experiments were carried out three times in a stirred ball mill (PE 075, Netzsch, Selb, Bavaria, Germany) at 20 °C (temperature control via a jacket with thermofluid) with 2 mm grinding beads and a three-disc stirrer with a maximum diameter of 6.4 mm. The total process time was 15 h. Therefore, two series of tests were carried out.

For the first set of experiments, a PTFE stirrer, self-built PTFE grinding chambers and “virgin” grinding beads were used to ensure that any inorganic particles found in the comminution product must originate from the grinding media. The stirring speed was adjusted to the density of the grinding media (see Table 1) to apply the same stress energies (*SE_max_* = 0.9 mJ) in the experiments. For the PBT product powders obtained at equal stressing conditions with respect to *SE_max_* and process time t, optical and thermal properties were analysed.

The second set of experiments was performed with the standard ceramic stirrer and a ceramic grinding chamber, both made of yttrium-stabilised zirconia. The grinding media used were the virgin grinding media from set one after they had been stressed for 15 h and cleaned. A fixed stirrer speed of 2000 min^−1^ was used to investigate the comminution behaviour of the PBT particles depending on the different grinding bead densities (*SE_max_* = 0.9–2.8 mJ, fixed SN).

### 3.3. Characterization

Laser diffraction particle sizing

Particle size distributions of the wet ground polymers were measured five times by laser diffraction (Mastersizer 2000/Hydro 2000 S, Malvern Panalytical, Malvern, United Kingdom) and an average was calculated. Small amounts of sodium dodecyl sulphate (Merck, 98% SD, Darmstadt, Hesse, Germany) were used to reduce surface tension and ensure stable dispersion during the measurements.

### 3.4. Scanning Electron Microscopy (SEM)

The scanning electron microscope GEMINI SEM 500 (Carl Zeiss AG, Oberkochen, Baden-Wuerttemberg, Germany) equipped with an SE2 detector operated at an accelerating voltage of 1 kV was used to evaluate the morphology and surface of the polymer particles. For this purpose, the polymer powder was immobilised on sticky carbon pads.

Energy dispersive X-ray (EDX) measurements were performed on an SEM ULTRATM 55 (Carl Zeiss AG, Oberkochen, Baden-Wuerttemberg, Germany) with a Noran System Six 302 Detector (Thermo Electron) and a maximum acceleration voltage of 10 kV.

### 3.5. Differential Scanning Calorimetry (DSC)

**Figure 3:**
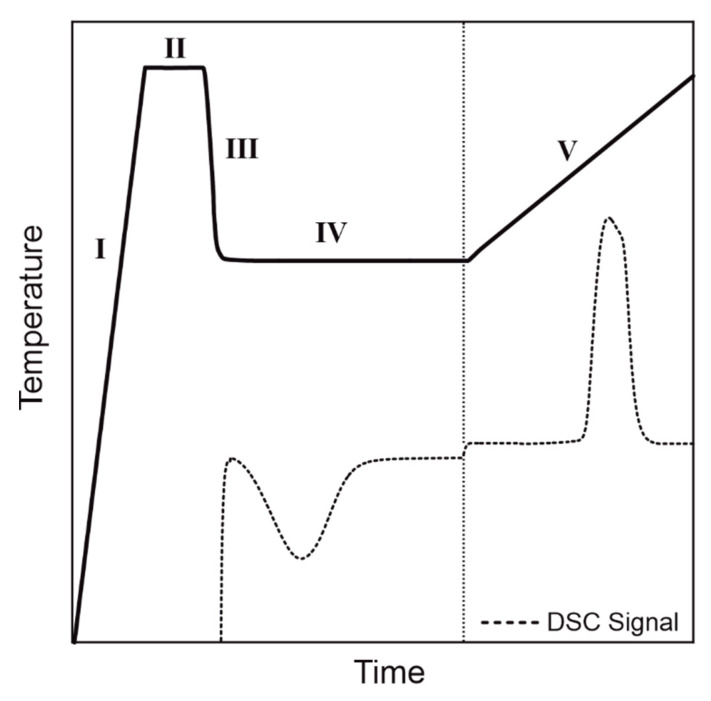
Schematic illustration of the used isothermal DSC program consisting of the 5 segments: heating above melting temperature (I), tempering (II), cooling to the isothermal temperature (III), isothermal holding step (IV) and melting of isothermal crystallised sample(V).

The thermal properties of the wet ground polymers were analysed by DSC (Polyma 214, Netzsch, Polyma 214, Selb, Bavaria, Germany). All samples (weight: 10 ± 0.1 mg) were placed in standard aluminum pans (concavus Lids (Al) NGB817526, Netzsch, Selb, Bavaria, Germany) with covers and measured in flushed dry nitrogen atmosphere. For dynamic measurements, the samples were heated and cooled from 20 °C to 260 °C at 10 K/min two times. For isothermal measurements, see Figure 3, the samples were heated up (100 K/min) to 260 °C (I) and tempered for 1 min to ensure complete melting of the sample (II). Next, the sample was quickly cooled down (−200 K/min) with no temperature dip to the isothermal temperature of 209 °C (III) and held there for 120 min (IV). Finally, the sample was heated up (10 K/min) to 260 °C (V) to evaluate the melting behaviour of the isothermally crystallised sample.

### 3.6. Thermogravimetric Analysis (TGA)

TGA measurements were performed using the Q50 (TA Instruments, New Castle, United Kingdom) under synthetic air atmosphere. The samples were heated to 900 °C at 50 K min^−1^ and held for 10 min. The residual mass corresponds to the inorganic content of the plastic powder.

### 3.7. X-ray Diffraction (XRD)

Diffractograms were measured with an Empyrean series 2 (Malvern Panalytical, Malvern, United Kingdom) in Bragg–Brentano geometry with a GaliPIX3D detector, Cu Kα radiation (154 pm, 40 kV, 40 mA), a step size of 0.014° and a scanning rate of 25 s/step within a range of 5° ≤ 2θ ≤ 100°. A flat layer of polymer powder was prepared on the sample holders and spun with a spinner revolution time of 0.5 s during the measurement.

### 3.8. Gel Permeation Chromatography (GPC)

For GPC measurements, the polymer was dissolved in hexafluoroisopropanol (HFIP) at a concentration of 0.002 g/mL. In order to prevent the association of polymer molecules due to dipole–dipole interactions, potassium trifluoro acetate was added to the solvent (0.05 mol/L) [37]. The solutions were analysed using triple detection GPC (TDA 305, Malvern Panalytical, Malvern, United Kingdom) with refractive index, viscometer and light scattering detector at room temperature using a flow-rate of 0.8 mL/min. Molar masses *M_w_* were calculated from light scattering using a refractive index increment of 0.221 mL/g.

### 3.9. UV-Vis Remission Spectroscopy

Optical properties of the polymer powders in the spectral range of 400 to 1000 nm were analysed by means of remission spectroscopy using a Lambda 950 UV/Vis spectrometer (Perkin Elmer, Rodgau, Hesse, Germany) with a 150 mm integrating sphere module from. The powders were prepared in a glass cuvette for the remission measurements. A light trap for 0 % reflectance and a Zenith polymer diffuse reflectance standard (SphereOptics GmbH, Ammersee, Bavaria, Germany) for 100 % reflectance were used as calibration.

## 4. Results

### 4.1. Comminution Kinetics of Polybutylene Terephthalate

**Figure 4:**
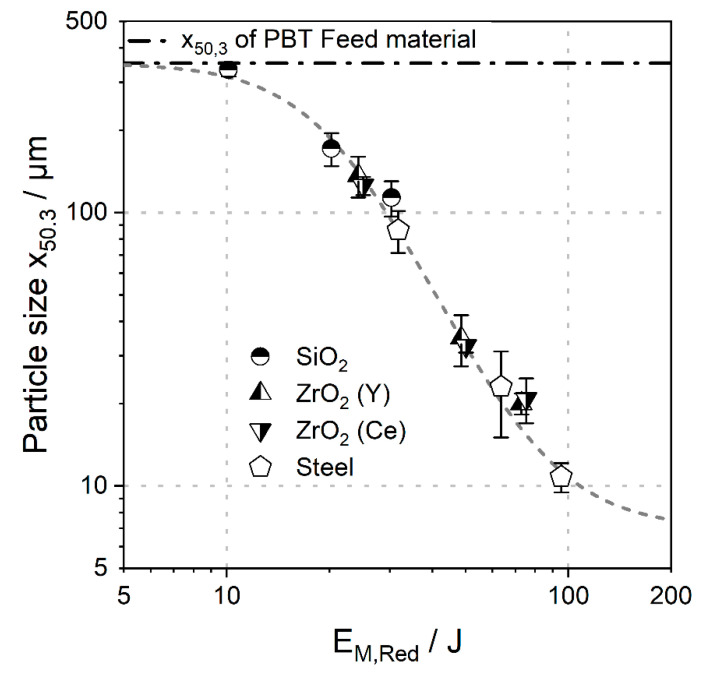
Double logarithmic plot of mean particle size *x*_50,3_ as a function of reduced energy input with standard deviation. Average of three measurements and standard deviation are shown.

For assessment of the comminution behaviour of PBT, 20 g of the thermoplast was ground in 180 g ethanol for 15 h with four different grinding media of 2 mm size (diameter). The grinding media volume was kept constant at 450 mL to ensure that a comparable number of grinding beads was used in each experiment (fixed SN). The results can be seen in Figure 4, where the mean particle size *x*_50,3_ is plotted against the reduced energy input (Equation (6)). A logistic function in the form of Equation (11) was fitted to the data as a sigmoidal trend between the feed particle size, and the grinding limit was observed in literature [31,32]. The mass median particle diameter *x*_50.3_ of the feed of 351.7 µm was set as the upper limit (*A*_1_). The lower limit (*A*_2_) was set at 7 µm, as this was the minimum achievable *x*_50.3_ in preliminary comminution experiments of PBT with 2 mm yttrium-stabilised zirconium oxide grinding media at 20 °C and 2000 rpm (*SE_max_* = 2.2 mJ). Densities of the different grinding beads are listed in Table 1.

(11) $$\frac{E_{M,Red}}{J}=\frac{A_{2}}{µm}+\frac{\frac{A_{1}}{µm}-\frac{A_{2}}{µm}}{1+{\left(x_{50.3}/x_{0}\right)}^{p}}=7+\frac{351.7-7\;}{1+{\left(x_{50.3}/x_{0}\right)}^{p}}$$

As can be seen, the mean particle size *x*_50.3_ follows the logistic function in dependency of the reduced specific energy *E_M,Red_* with a center *x*_0_ of 20.7 ± 0.5 µm and a power *p* of 2.9 ± 0.2. For all tested grinding media, a critical stress energy must be overcome for size reduction to take place. Then, a specific particle size can be achieved by adjusting energy input and process time. For a fixed process time, grinding beads with a higher density produce smaller product particles, since a higher stress energy (*SE_max_* = 0.9–2.8 mJ) is applied at the same stress number (Equation (4)). For example, after 15 h of wet comminution, the product particles obtained from grinding with glass beads (*x*_50,3_ of 137 µm) are larger than the powders ground with higher density grinding media such as steel (*x*_50,3_ of 9 µm) or yttrium-stabilised zirconium oxide beads (*x*_50,3_ of 21 µm). Schmidt et al. reported similar trends for the comminution behaviour of polystyrene under comparable process conditions (2 mm yttrium-stabilised zircon grinding beads, *T* = 20 °C, *t* = 15 h, *SE_max_* = 2.6 mJ). Product particles as small as *x*_50.3_ ~ 2 µm were obtained, due to the higher brittleness of (amorphous) polystyrene compared to PBT [15].

### 4.2. Intrinsic Properties of Wet Ground PBT

For the analysis of thermal and optical properties, as well as the crystalline structure of wet ground PBT, the powders of the first set of experiments were used, where a uniform stress energy of *SE_max_* = 0.9 mJ was set by adjusting the stirrer speed to the density of the grinding media (Table 1). Due to the PTFE grinding chambers, the friction behaviour between the grinding media and the grinding chamber has changed and a larger drum has formed. To prevent overflowing, the experiments were scaled down to 400 mL of grinding media, 160 g of EtOH and 17.6 g of PBT.

#### 4.2.1. Optical Properties of Wet Ground PBT

**Figure 5:**
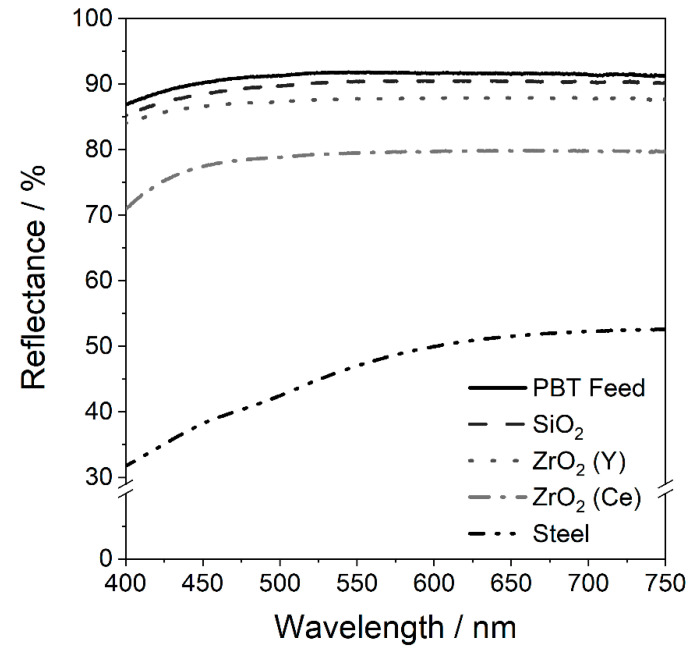
Reflectance spectra of PBT powders comminuted with different grinding beads (*SE_max_* = 0.9 mJ, 20 °C, 15 h) in the visible region. Mean value from three experiments is shown.

The influence of the used grinding beads on the reflectance of the wet ground powder is depicted in Figure 5.

The remission spectra of the PBT powders show the discolouration after wet comminution, which is caused by abrasion of the grinding media that can be found on the surfaces of the PBT particles. White powders can be obtained when using glass or yttrium-stabilised zirconia grinding media. Cerium-stabilised zirconia grinding media result in a slightly greyish hue and PBT powders ground with steel beads show a distinct brownish rust-coloured discolouration. Since white powders are favoured for the PBF-LB/P process, as finished parts can be dyed more easily in a subsequent step, the glass and yttrium-stabilised zirconia grinding media are best suited in terms of optical properties.

#### 4.2.2. Analysis of the Abrasion

**Figure 6:**
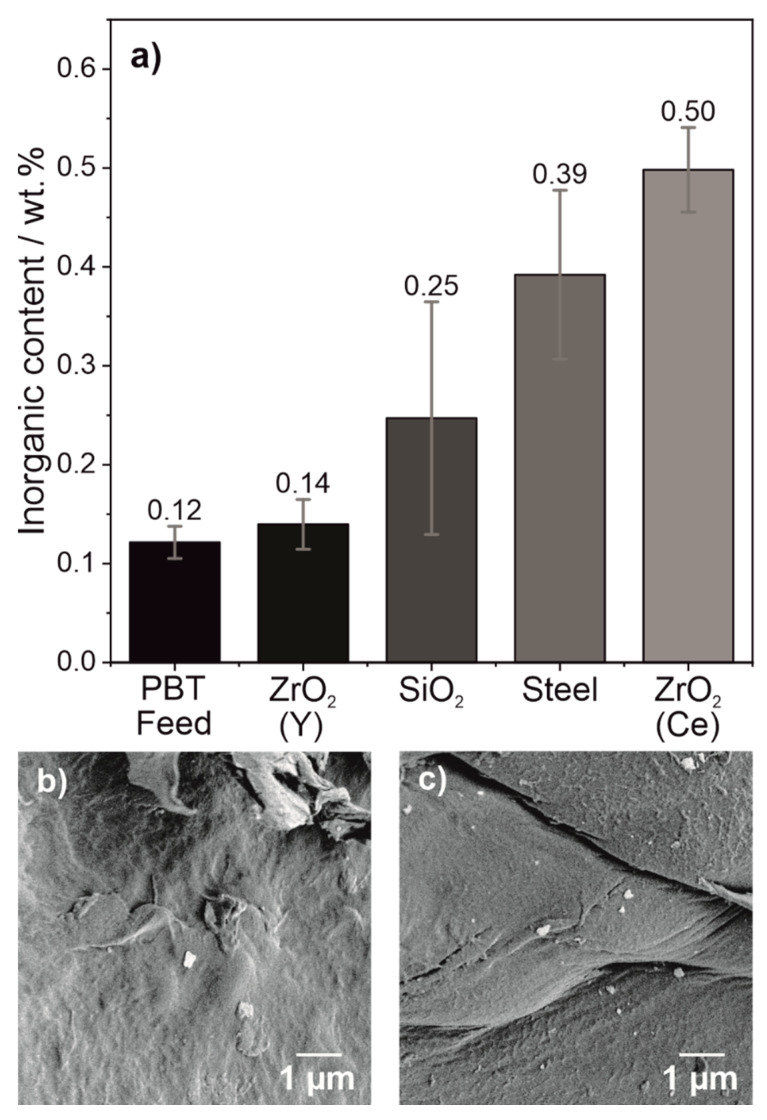
Inorganic content of (**a**) wet ground PBT particles (*SE_max_* = 0.9 mJ, 20 °C, 15 h) using different grinding media as determined by TGA with representative SEM images of the abrasion on the PBT particle surface comminuted with (**b**) ZrO_2_ (Y) and (**c**) ZrO_2_. Average of three measurements and standard deviation are shown.

Thermogravimetric analysis of the ground PBT powder was performed to quantify the amount of abrasion in the powders. The results are summarized in Figure 6.

TGA measurements show that the PBT feed material already has an inorganic content of 0.12 ± 0.02 wt.%. This is most likely inorganic added to the polymer by the manufacturer. The PBT powder wet ground for 15 h with ZrO_2_ (Y) shows the lowest inorganic content with 0.14 ± 0.03 wt.%, which is indistinguishable from the abrasion content of the feed material within the scope of the standard deviation. Therefore, the powders comminuted with ZrO_2_ (Y) grinding media are the most suitable for the PBF-LB/P in terms of abrasion quantity. SiO_2_, steel and ZrO_2_ (Ce) grinding media produced a product with higher abrasion content of 0.25 ± 0.12 wt.%, 0.39 ± 0.09 wt.% and 0.50 ± 0.04 wt.% respectively. In the SEM images (Figure 6b,c), it can be observed that the abrasion from the grinding media is homogeneously distributed onto the surface of the PBT particles.

The higher abrasion content compared to other studies [38,39] is due to the fact that virgin grinding beads ‘as received’ without any pre-conditioning treatment were used. A widely used model to describe product reliability is the bathtub model [40]. Especially at the beginning, products show a high failure rate, often referred to as infant mortality. Since virgin grinding beads were used to avoid possible external contamination, there is also more abrasion compared to already used grinding beads. Thus, the results such as altered crystallisation, which scale with the amount of abrasion, can be considered worst-case scenarios.

#### 4.2.3. Diffractograms of Wet Ground PBT

**Figure 7:**
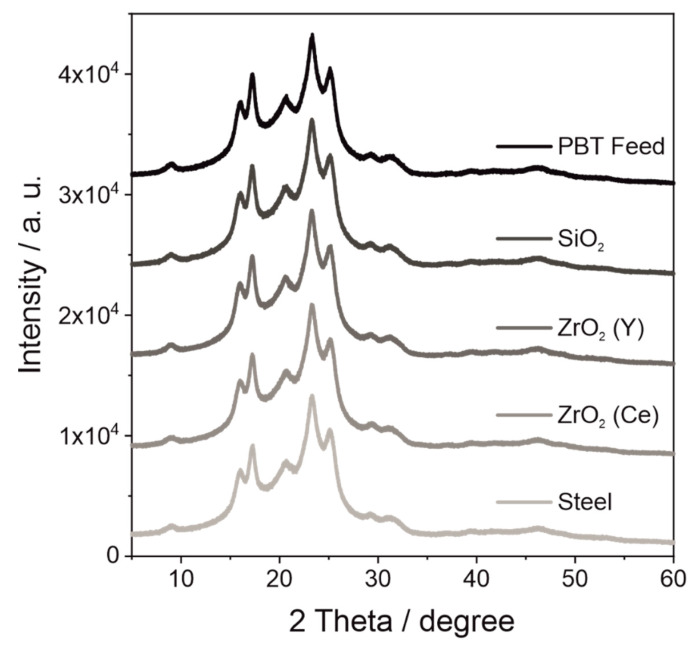
Diffractograms of PBT wet ground with different grinding bead materials (*SE_max_* = 0.9 mJ, 20 °C, 15 h).

In the case of PET, comminution structural changes (amorphization) caused by mechanical milling could be observed in diffractograms. [41] Therefore, XRD measurements of the PBT feed material and the comminuted powders (Figure 7) were performed in order to exclude changes in the crystal structure.

From the diffractograms of the differently comminuted PBT powders, obviously the crystal structure remains unchanged during wet comminution. PBT feed material as well as the product powders show the typical peaks for α-PBT at 2 theta values of 16.2°, 17.3°, 20.6°, 23.3° and 25.1°, which can be assigned to the (011), (010), (102), (100) and (111) planes, respectively [20,42]. In the wet grounded PBT powder, the amount of abrasion was too small to become recognizable in the XRD pattern. Likewise, in FTIR measurements, no changes in spectrum could be observed between the crushed PBT and the PBT feed material (Supplementary Information S2). Therefore, EDX measurements were carried out for these powders. The inorganic particles on the surface of the PBT could be identified as the grinding media material (Supplementary Information S3). In the case of the PBT powder, which was wet ground with SiO_2_ and ZrO_2_ (Y) media, the powder was first incinerated and the ash was analysed, as the amount of abrasion in these samples was very low.

#### 4.2.4. Thermal Properties of Wet Ground PBT

**Figure 8:**
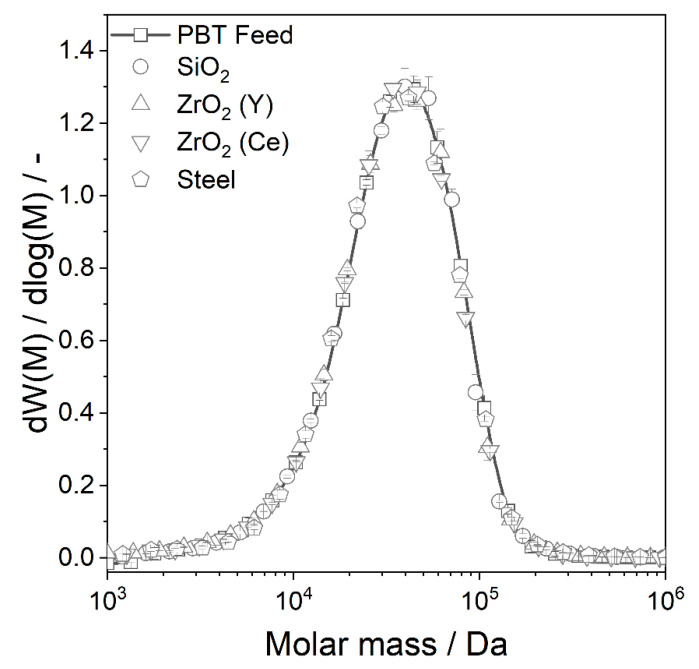
Molar mass distribution of PBT wet ground with different grinding bead materials (*SE_max_* = 0.9 mJ, 20 °C, 15 h).

Many parameters influence the thermal properties of polymers such as crystal structure, the presence of (heterogeneous) nuclei or the chain length of the polymer. In order to be able to assign the changed thermal properties to one parameter, the other parameters must be excluded. A change in the crystal structure of the PBT was excluded in Section 4.2.3. By means of gel permeation chromatography (GPC), it could be shown that the molar mass distribution of the polymer does not change significantly during wet comminution (Figure 8). Thus, the wet comminution process is suitable for producing PBT microparticles without noticeably damaging the polymer structure. Furthermore, preliminary tests on mixing PBT with ethanol and stirring for 15 h had no influence on the thermal properties (e.g., by washing out additives). By blending the PBT with the PTFE of the grinding chamber (potential abrasion of the grinding chamber walls), a shift of the crystallisation peak to lower temperatures could be observed (Supplementary Data S1).

In conclusion, any changes of the crystallisation peak to higher temperatures (accelerated crystallisation kinetics) can be attributed to the abrasion of the grinding beads, which serve as heterogeneous nuclei during crystallisation and, thus, accelerate nucleation.

##### Dynamic DSC

**Figure 9:**
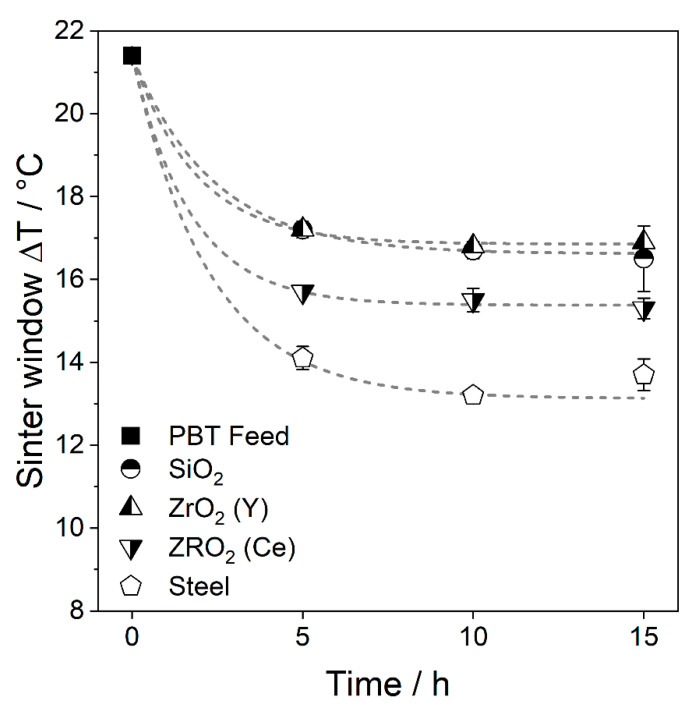
Sintering window widths of the wet-comminuted PBT powders (*SE_max_* = 0.9 mJ, 20 °C) with exponential fit. Average of three measurements and standard deviation are shown.

The width of the sintering window, which is defined as the temperature range between the onset of the melting point and the onset of the crystallisation peak, was evaluated by DSC measurements (Figure 9). No influence of the wet comminution on the melting peak position could be observed (Thermograms in Supporting Information Figures S4 and S5). Therefore, the changes of the thermal process window width are due to a shift of the crystallisation peak.

All wet ground PBT powders show a reduction in the sintering window. The greatest changes can be observed in the first 5 h, afterwards a plateau is reached. The PBT powder, which was comminuted for 15 h with steel grinding beads, shows the smallest sintering window of 13.7 ± 0.4 °C, followed by ZrO_2_ (Ce) SiO_2_ and ZrO_2_ (Y) with a sintering window width of 15.3 ± 0.3 °C, 16.5 ± 0.8 °C and 16.9 ± 0.1 °C, respectively. The change in the sintering window can be explained by the formation of nano-sized abraded particles during wet comminution This fraction increases with an increasing comminution time and the fragments serve as heterogeneous nuclei during nucleation, which reduce the nucleation barrier locally. At a certain point, sufficient heterogeneous nuclei are present to trigger heterogeneous nucleation throughout the sample. Consequently, further addition of heterogeneous nuclei will not further accelerate the nucleation. If crystal growth is not influenced by the heterogeneous nuclei, a plateau of the thermal data such as sintering window width, *t_Peak_* or *t*_1/2_ occurs. Therefore, the nucleation rate depends on the available heterogeneous interface and the nucleation efficiency of that interface [43]. Assuming the resulting debris has similar particle size distributions, the available surface area scales linearly with the abrasion mass. Thus, steel must have a greater nucleation efficiency compared to ZrO_2_ (Ce), as faster crystallisation kinetics was observed at a lower abrasion fraction.

##### Isothermal DSC

**Figure 10:**
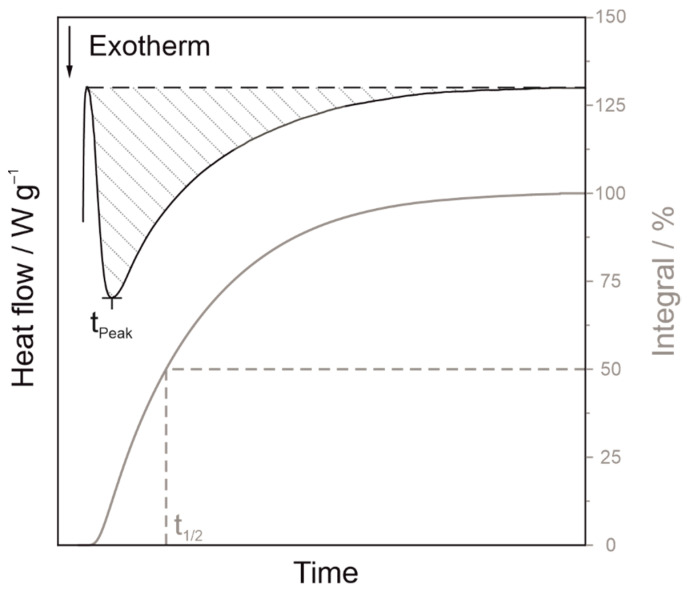
Schematic representation of the crystallisation halftime *t*_1/2_ and the time to reach the crystallisation peak *t_Peak_*.

**Figure 11:**
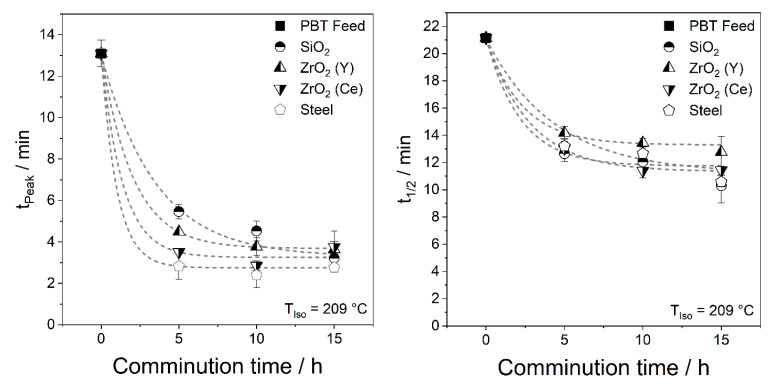
Time to reach crystallisation maximum *t_Peak_* and time to reach 50% crystallisation *t*_1/2_ for wet ground PBT (*SE_max_* = 0.9 mJ, 20 °C) with different grinding bead materials. Average of three measurements and standard deviation are shown.

Isothermal DSC data were analysed using the time to reach the crystallisation peak *t_Peak_* and the time, at which half crystallisation is complete, *t*_1/2_, as schematically shown in Figure 10 for a non-Gaussian peak. A horizontal baseline was used in the calculation of the crystallisation area. For fast-crystallising samples, such as PBT wet ground with steel grinding media, the transient signal had to be subtracted as described in [44], see Figure 11.

Similar to the dynamic measurements, *t_Peak_* and *t*_1/2_ decrease with increasing comminution time due to the resulting abrasion until a plateau is reached. Therefore, *t_Peak_* shortens significantly more than *t_1/2._* Since nucleation occurs at the beginning of crystallisation, the effect of altered nucleation is more apparent in *t_Peak_*. For a symmetrical peak, *t_Peak_* and *t*_1/2_ are identical. If heterogeneous nuclei accelerate nucleation, but crystal growth remains unchanged, tailing of the peak will occur as shown in Figure 10. For example, *t*_1/2_ and *t_Peak_* of the powder wet ground with ZrO_2_ (Y) drop within the first 5 h from 22.0 min and 13.6 min to 14.25 min and 5.0 min, respectively. Subsequently, the acceleration of crystallisation flattens out and the values *t*_1/2_ and *t_Peak_* reach 13.5 min and 4.2 min after 15 h. In other words, for ZrO_2_ (Y) grinding media, t_1/2_ was reduced by 39 % and*t_Peak_* was reduced by 69 % after 15 h milling, indicating that nucleation is strongly accelerated, but crystal growth is hardly affected.

**Figure 12:**
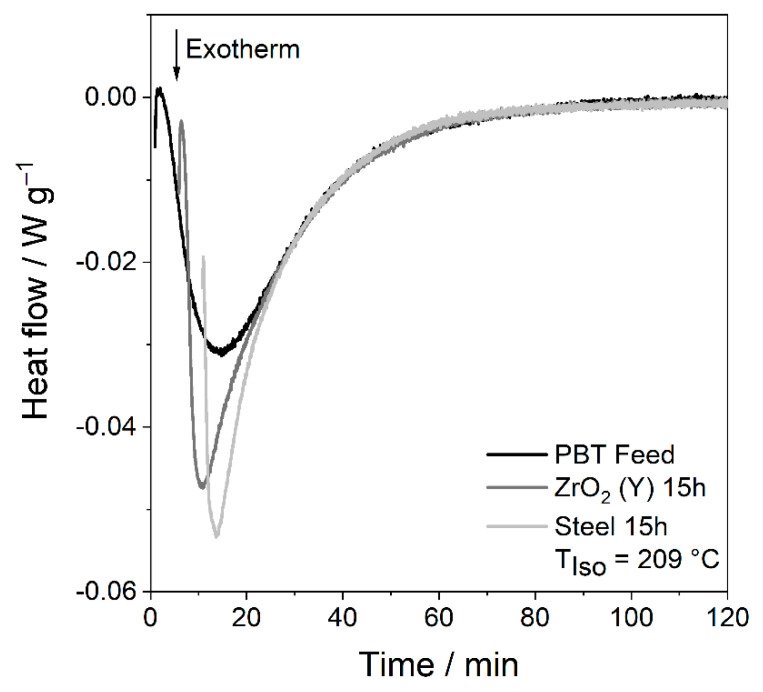
Isothermal crystallisation at 209 °C of PBT feed material and wet ground powder with steel and ZrO_2_ (Y) grinding beads (*SE_max_* = 0.9 mJ, 20 °C, 15 h), where wet ground samples have a time off-set.

In addition, Figure 12 shows the time course of the isothermal crystallisation of the PBT feed material and the wet ground PBT material with steel and ZrO_2_ (Y) after 15 h. Therefore, the course of the thermograms of PBT stressed with ZrO_2_(Y) and steel media has a given temporal off-set, trying to overlap the later part of the thermogram where the crystal growth happens. This nicely visualises the fact that nucleation is accelerated, but crystal growth is hardly affected. In the case of the wet ground PBT, the time to reach the peak is greatly reduced, as evidenced by the large temporal off-set needed. However, the thermogram behind the peak is almost identical with the thermogram of the PBT feed. This proves that in fact only the nucleation, which takes place in the early stage of the crystallisation process, is accelerated. The crystal growth, which determines the later course of the thermogram, remains unchanged. Similar trends were seen using SiO_2_, chromium steel and ZrO_2_ (Ce) grinding media (see Supporting Information Figures S6–S9).

**Figure 13:**
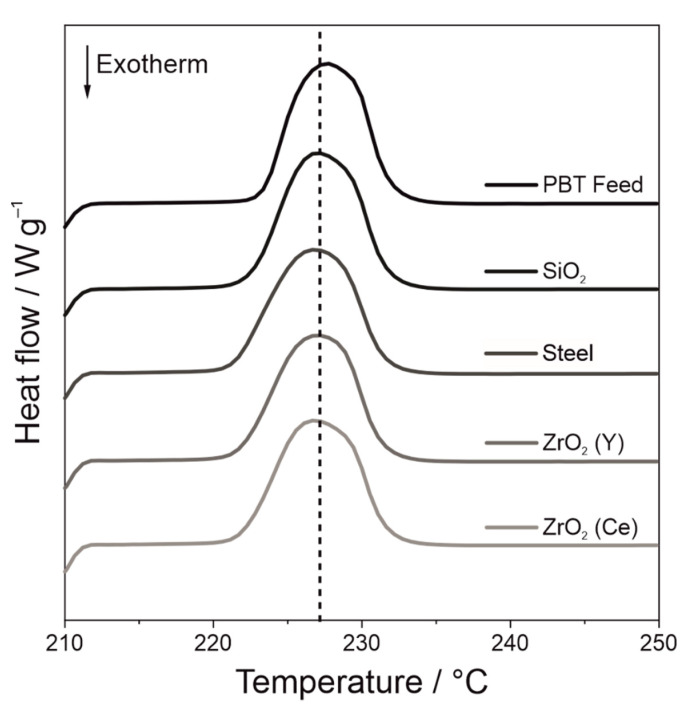
Melting of isothermally crystallised PBT samples at 209 °C wet ground with different grinding bead materials (*SE_max_* = 0.9 mJ, 20 °C, 15 h).

The melting of the isothermally crystallised samples (Figure 13) indicates that the same crystal structures have been formed after the melting, as melting takes place at the same temperatures. Therefore, an acceleration of the crystallisation due to different forming crystal structures are unlikely and the accelerated crystallisation can be assigned to the abrasion acting as heterogeneous nuclei.

## 5. Conclusions

In this study, the cold wet comminution of PBT in stirred media mills was investigated using PBT. The comminution behaviour, optical properties and thermal properties of wet comminuted PBT powders with different grinding media materials were investigated in order to find optimised grinding media. Chrome steel grinding media showed the fastest comminution kinetics due to their high density. However, they are not suitable for the production of PBF powders due to the significant discolouration of the powder and the drastically accelerated melt crystallisation due to the debris content in the product. SiO_2_ grinding media showed comminution kinetics too slow to be economically interesting. Yttrium- and cerium-stabilised zirconia grinding media both showed advantageous comminution kinetics and any colour effects were not observed. The powders comminuted with ZrO_2_ (Y) showed less abrasion and, therefore, less accelerated crystallisation kinetics compared to ZrO_2_ (Ce). Consequently, yttrium-stabilised ZrO_2_ grinding media turned out to be the optimal grinding media for the wet comminution of polymers amongst the tested materials, as they showed fast comminution kinetics, no discolouration and the least altered thermal properties of the ground thermoplast in terms of dynamic and isothermal crystallisation. Our approach can be extended to commercially available powders for powder-based additive manufacturing.

## Figures and Tables

**Table 1:** Grinding media densities and agitator speeds used to achieve a stress energy of *SE_max_* = 0.9 mJ.

Grinding Media	SiO_2_	ZrO_2_ (Y)	ZrO_2_ (Ce)	Steel
ρ/g cm^−3^	2.5	6.0	6.2	7.9
n/min^−1^	2000	1290	1270	1130

## Data Availability

The data that support the findings of this study are available from the corresponding author, upon reasonable request.

## Supplementary Materials

The following supporting information can be downloaded at: https://www.mdpi.com/article/10.3390/polym14040810/s1.

## Nomenclature

*A*	Surface
*A*_1_	Initial Value
*A*_2_	Final value
*c_V_*	Solids concentration by volume
*d_GM_*	Diameter of the grinding media
*d_tip_*	Agitator diameter
*E_M_*	Mass-specific energy input
*E_M,Red_*	Reduced mass-specific energy input
*G_M_*	Change in free energy of melting
*M*	Elastic moduli
*n*	Agitator rotation speed
*N_C_*	Number of impacts between the grinding media
*N_P_*	Total number of particles in the medium
*p*	Power
*P_S_*	Probability that a particle is significantly stressed
*SE*	Stress energy
*SE_max_*	Maximal stress energy
*S_M_*	Change in entropy of melting
*SN*	Average number of stress events
*SN_RED_*	Reduced number of stress events
*t*	Process time
*t*_1/2_	Crystallisation halftime
*T_C_*	Crystallisation temperature
*T_M_*	Melting temperature
*t_Peak_*	Time to reach crystallisation peak
*V*	Volume
*v_tip_*	Speed of agitation
*x*_0_	Center
*x*_50.3_	Mean particle size by volume
*x_Feed_*	Characteristic particle size of the feed
$\varphi_{GM}$	Filling ratio
ε	Bulk porosity
$\rho_{GM}$	Density of the grinding media
$T_{M}^{0}$	Equilibrium melting temperature
σ	Specific surface free energy
Δ *g*	Melting free energy of unit volume
Δ *h*	Specific heat of fusion
Δ *H_M_*	Chance in heat of fusion
Δ *s*	Specific enthalpy of fusion
Δ *T*	Supercooling
