# Supplementary material for: Abrasion-Induced Acceleration of Melt Crystallisation of Wet Comminuted Polybutylene Terephthalate (PBT)
Source: Polymers (Basel). 2022 Feb 19;14(4):810. doi: 10.3390/polym14040810 (PMC8963030; doi:10.3390/polym14040810)
Supplement: Supplementary file 1 [file polymers-14-00810-s001.zip › polymers-1585206-supplementary.pdf]

1. Supporting Information

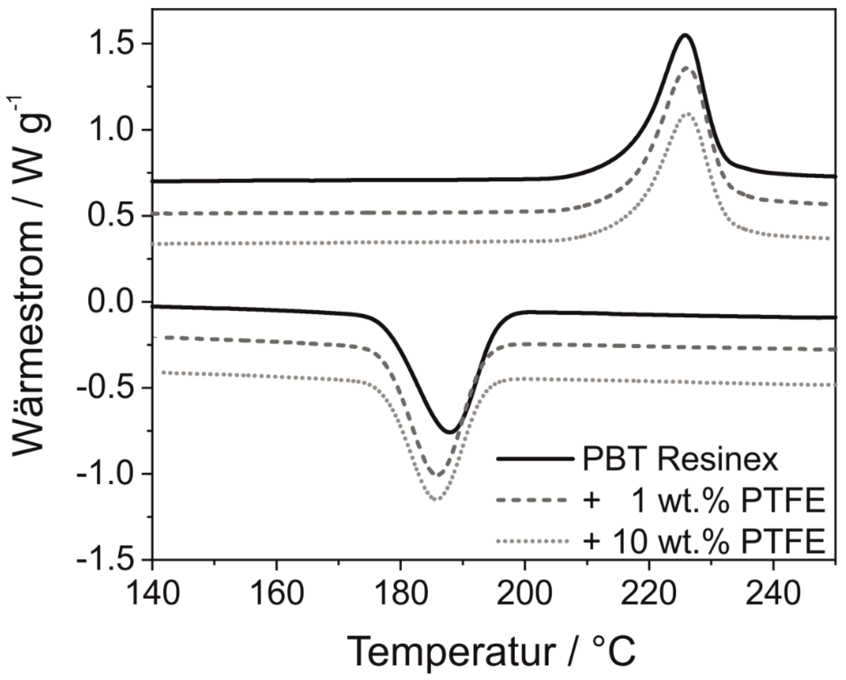

Figure S 1: Dynamic DSC thermograms of PBT feed materials and blended PBT with 1 wt.% and 10 wt.% PTFE of the grinding chamber. Blended PBT crystallises at lower temperatures (slower).

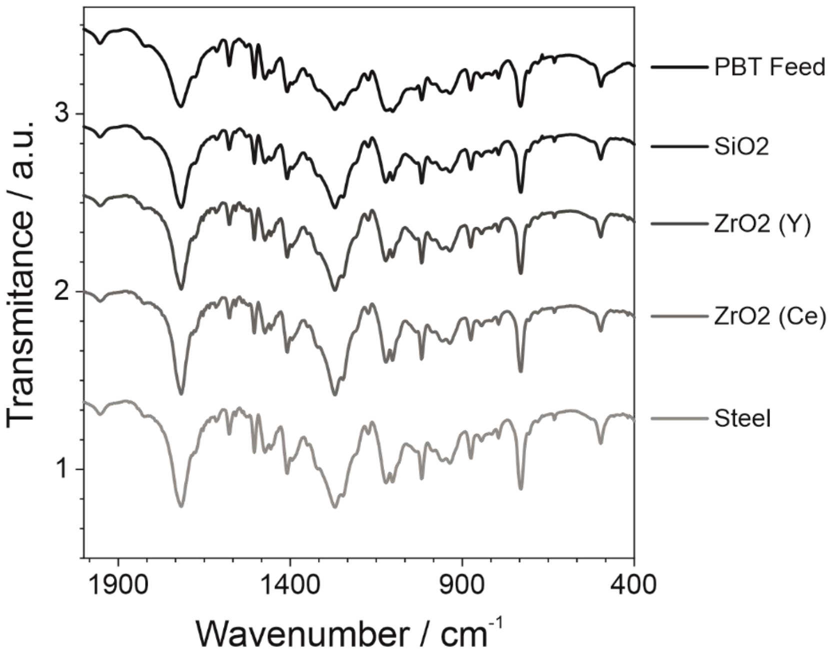

Figure S 2: FTIR Spectra (KBr Pellet Method) of wet ground PBT with different grinding bead materials. Mean value from three experiments are shown. Peaks of the PBT feed material can be found in all comminuted samples.

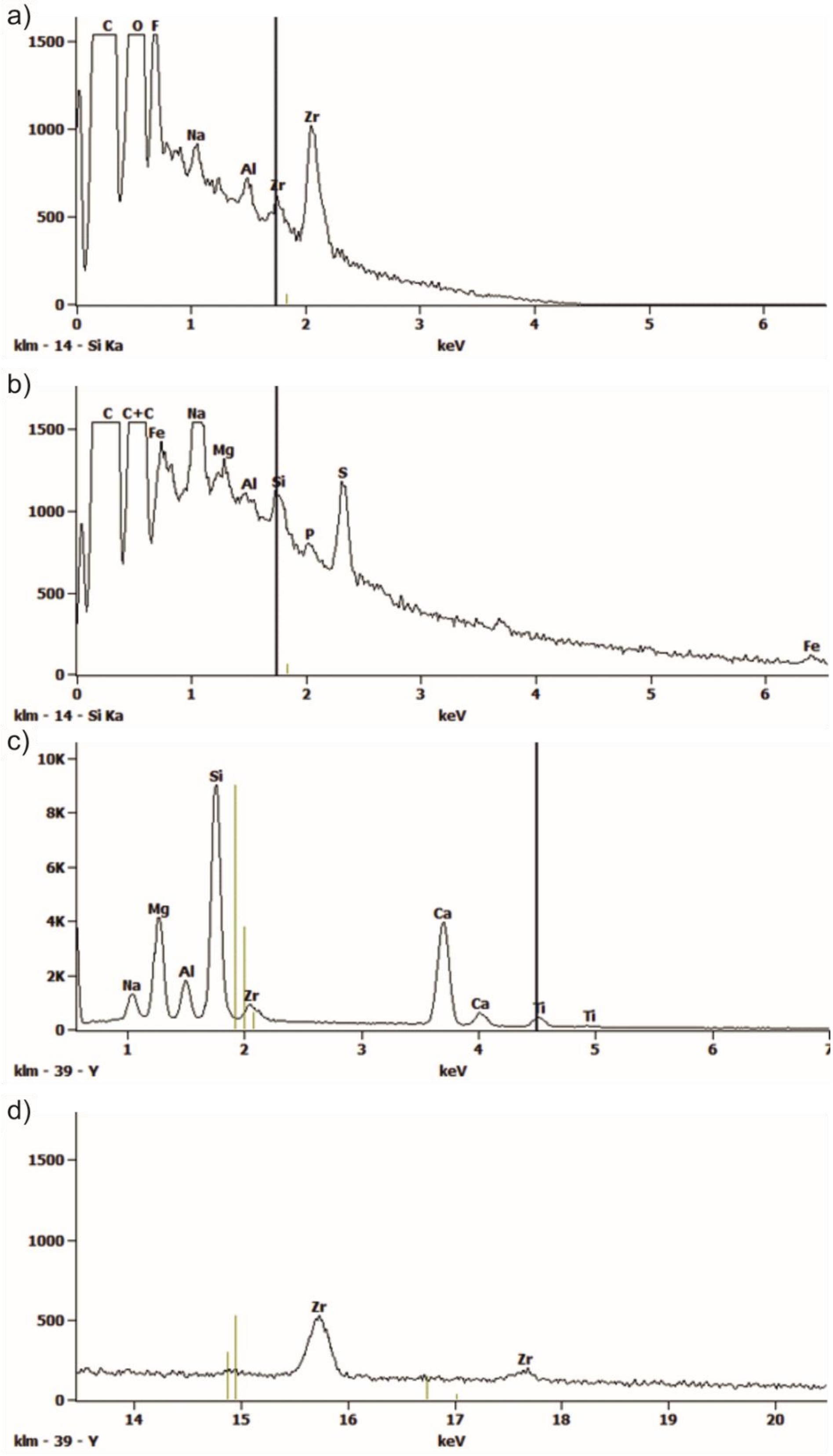

Figure S 3: EDX Spectrum of wet-grind PBT with grinding balls made of a) ZrO_2 (Ce), b) Steel, c) SiO_2 and d) ZrO_2 (Y). Due to the low amount of abrasion, the samples with c) SiO_2 and d) ZrO_2 (Y) were first ashed and then analysed.

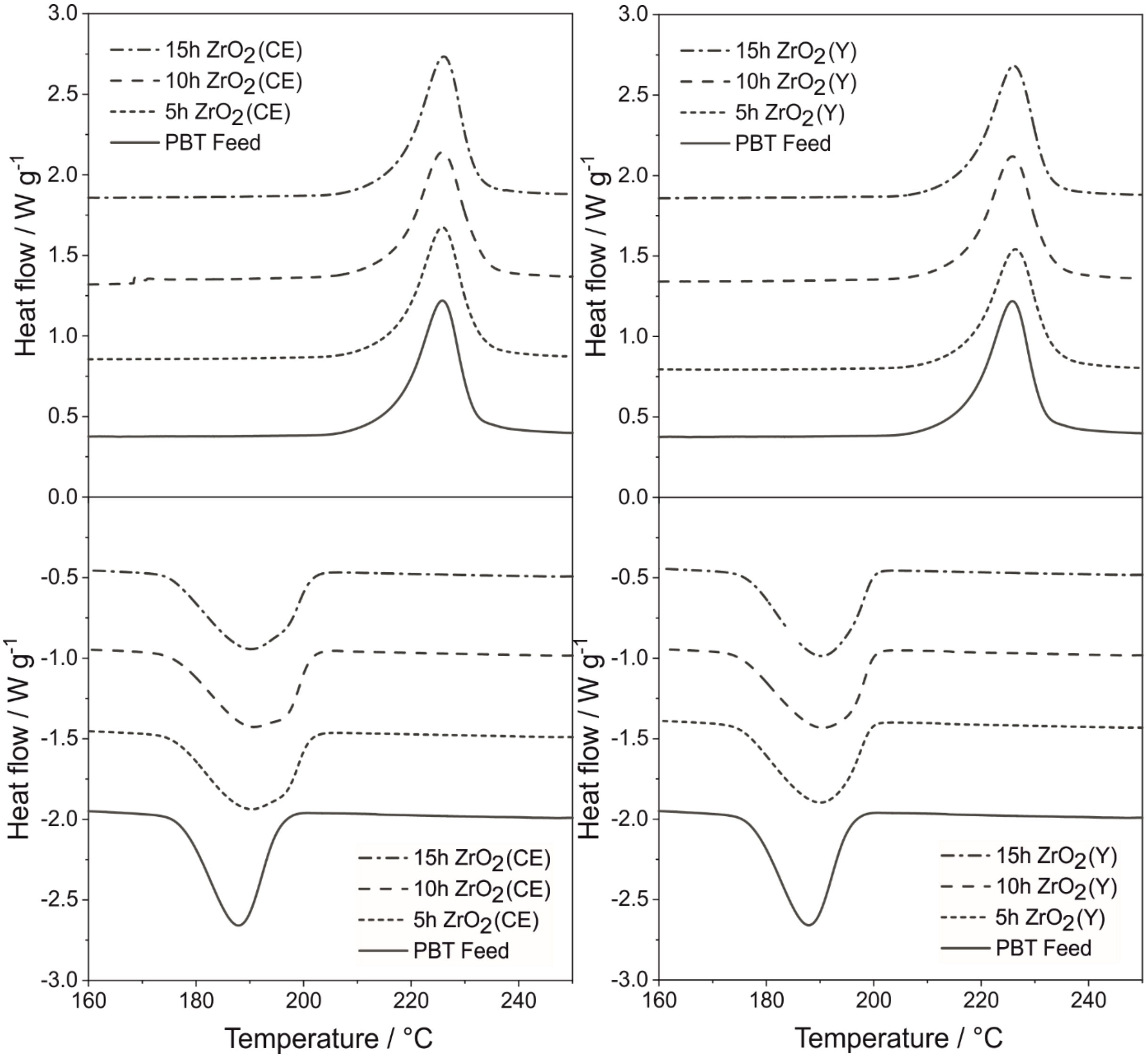

Figure S 4: Thermogram (10 K/min,1st Heat and Cool Cycle) of PBT Feed Material and wet ground PBT with yttrium and cerium stabilised zirconia grinding media (SE_{Max} = 0,9 mJ, 20 °C) at different grinding times.

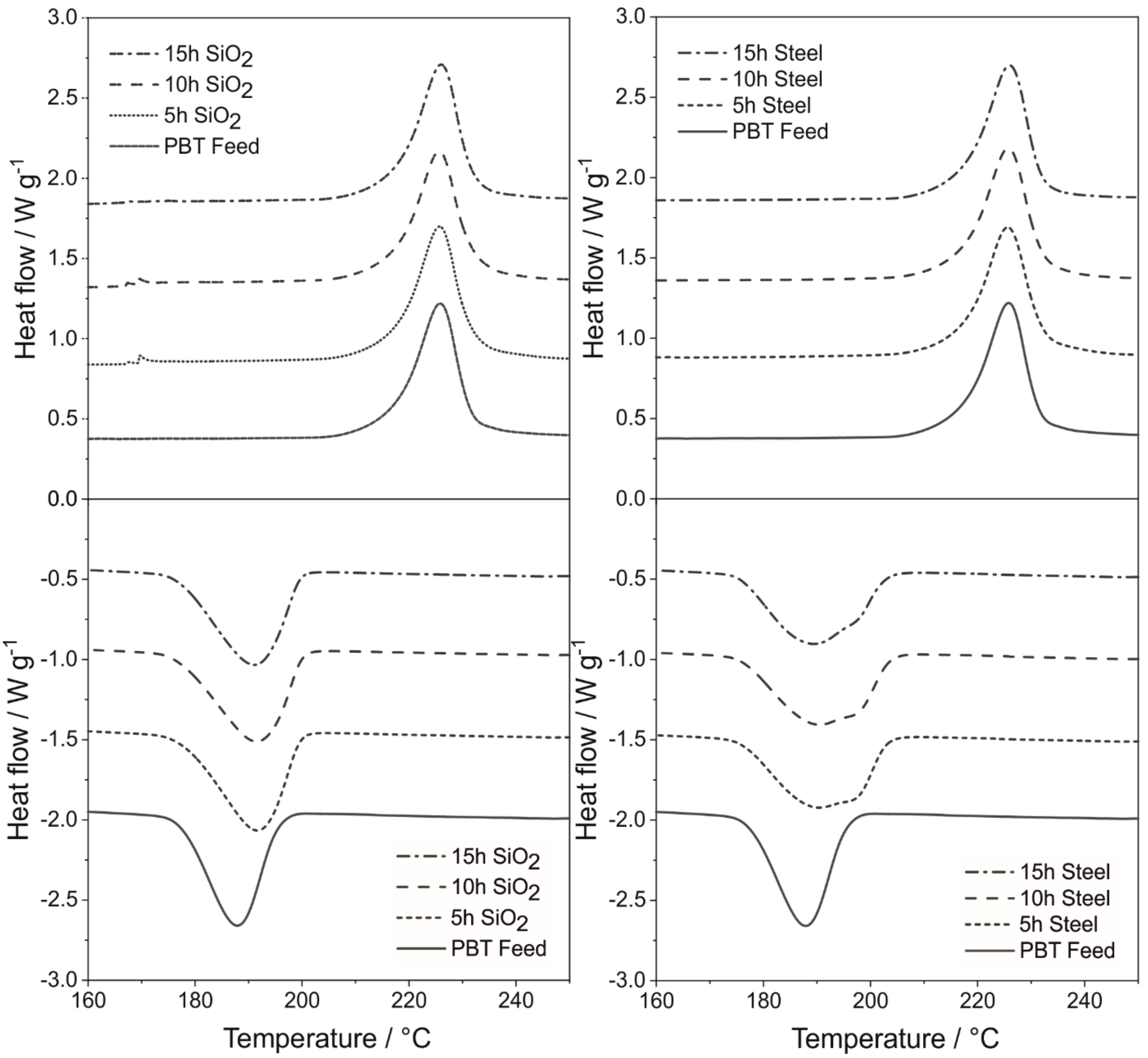

Figure S 5: Thermogram (10 K/min,1st Heat and Cool Cycle) of PBT Feed Material and wet ground PBT with glass and steel grinding media (SE_{Max} = 0,9 mJ, 20 °C) at different grinding times.

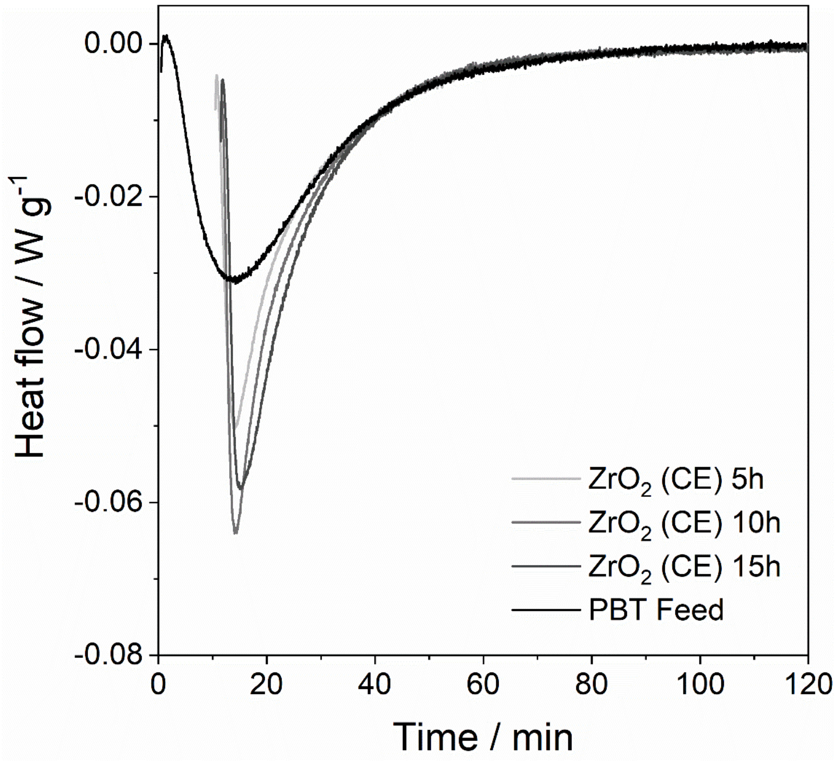

Figure S 6: Isothermal crystallisation at 209 °C of PBT feed material and wet ground powder with cerium stabilised zirconia grinding media (SE_{max} = 0.9 mJ, 20 °C) at different grinding times, where wet ground samples have a time off set.

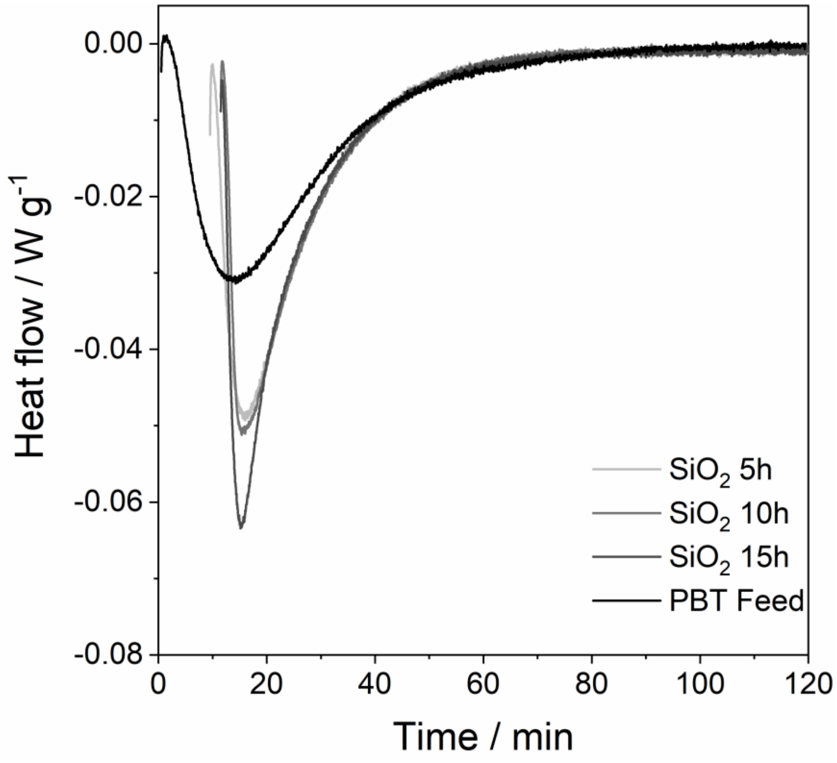

Figure S 7: Isothermal crystallisation at 209 °C of PBT feed material and wet ground powder with glass grinding media (SE_{max} = 0.9 mJ, 20 °C) at different grinding times, where wet ground samples have a time off set.

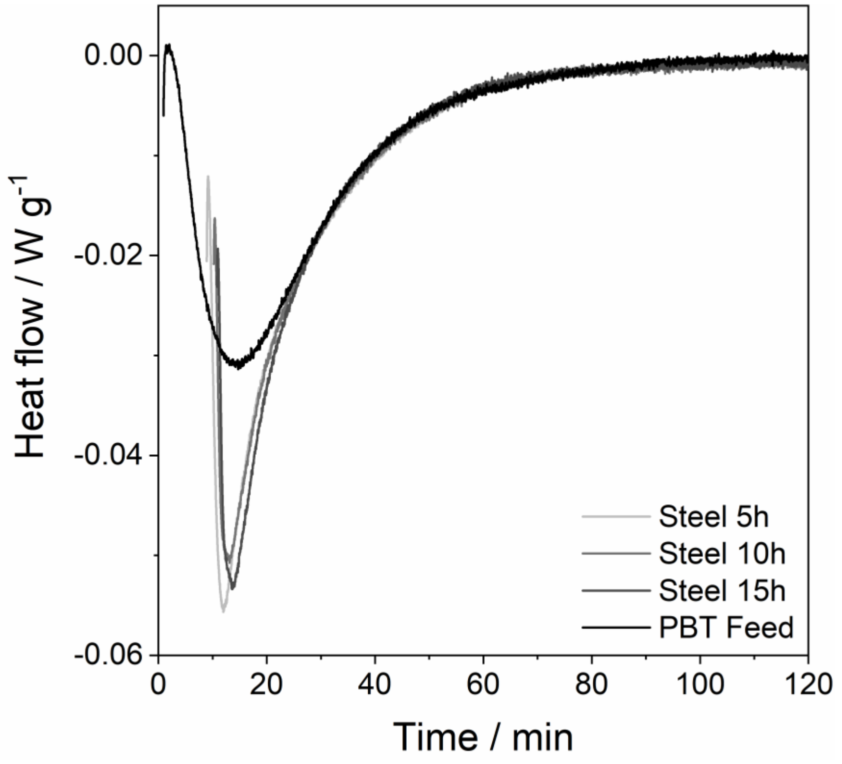

Figure S 8: Isothermal crystallisation at 209 °C of PBT feed material and wet ground powder with steel grinding media (SE_{max} = 0.9 mJ, 20 °C) at different grinding times, where wet ground samples have a time off set.

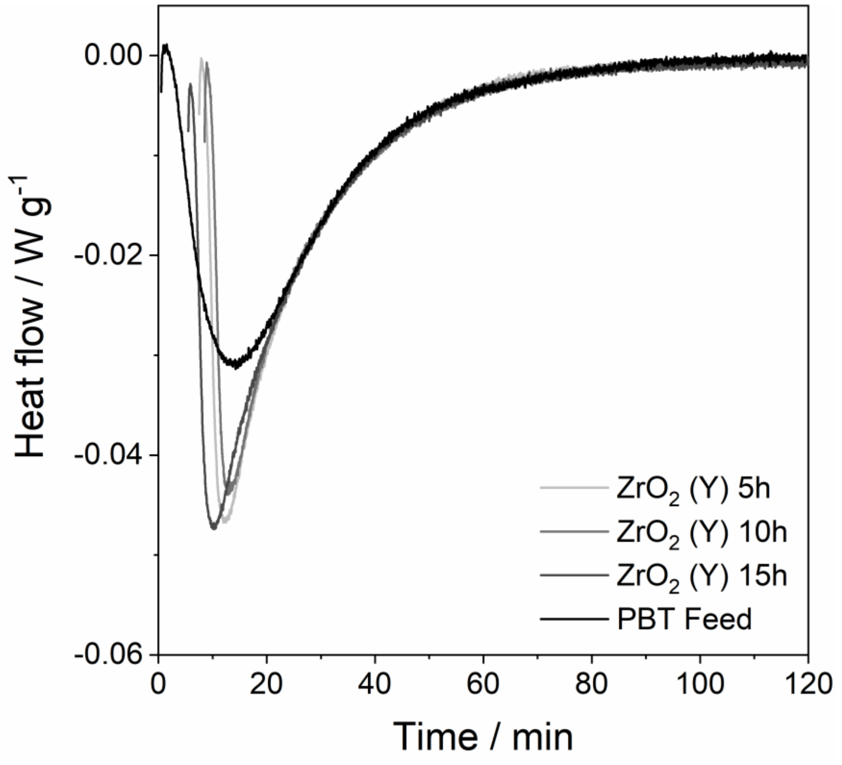

Figure S 9: Isothermal crystallisation at 209 °C of PBT feed material and wet ground powder with yttrium stabilised zirconia grinding media (SE_{max} = 0.9 mJ, 20 °C) at different grinding times, where wet ground samples have a time off set.
